# Ambulatory blood pressure monitoring in Egyptian children with nephrotic syndrome: single center experience

**DOI:** 10.1186/s13052-024-01775-x

**Published:** 2024-10-12

**Authors:** Eman Abobakr Abd Alazem, Sonia Ali El-Saiedi, Shradha Chitrakar, Shorouk A. Othman

**Affiliations:** 1 Division of pediatric nephrology, Department of Pediatrics, Kasr Alainy Faculty of Medicine, Cairo University, Cairo, Egypt; 2 Division of pediatric cardiology, Department of Pediatrics, Kasr Alainy Faculty of Medicine, Cairo University, Cairo, Egypt

**Keywords:** Ambulatory blood pressure, Masked hypertension -nephrotic syndrome

## Abstract

**Background:**

Hypertension (HTN), especially masked hypertension, is one of the cardiovascular consequences of nephrotic syndrome. Masked hypertension cannot be identified during routine follow-up visits and adversely effects the patients’ cardiac function. The purpose of this study was to use ambulatory blood pressure monitoring (ABPM) to evaluate the blood pressure status of children with nephrotic syndrome.

**Methods:**

Ninety children with nephrotic syndrome (NS) participated in this cross-sectional study, which was carried out at Cairo University Children Hospital’s nephrology clinic (CUCH). A sphygmomanometer was used in the clinic to measure blood pressure, and a Meditech monitor was used for 24-hour ambulatory blood pressure monitoring (ABPM). Interventricular septum (IVS) was measured, and heart functions were evaluated, using echocardiography.

**Results:**

Two groups comprised the included patients: Group1 (*n* = 70): HTN group included masked and ambulatory hypertension, and Group 2 (*n* = 20): non-HTN group included normal blood pressure, white coat HTN and well controlled HTN, 35% of the studied cohort (*n* = 32/90) had masked HTN.The serum urea was significantly higher in HTN group than non-HTN group with *p*-value: 0.047, while the serum albumin was significantly lower in HTN group than non-HTN group with *p*-value: 0.017. The cut-off point of 9.9, the sensitivity and specificity of serum urea to predict the occurrence of hypertension in NS patients was 92.9% and 35% respectively, with *p*-value : 0.024 and 95% CI (0.534–0.798). The z score of IVS is significantly higher in group 1 (2.5 ± 1.2) when compared to group 2 (1.7 ± 2.1) with *p*-value: 0.025 and Among group 1, it was noticed that 74% (*n* = 52/70) of them were systolic non-dipper, also it was observed that the mean serum potassium and cholesterol were significantly higher among systolic non-dipper when compared with systolic dipper patients with *p*-values: 0.045 and 0.005 respectively.

**Conclusion:**

Children with nephrotic syndrome are particularly vulnerable to experience ambulatory hypertension and masked hypertension, which may adversely impact their cardiac condition because they are not detectable by standard blood pressure readings at the clinic.

**Supplementary Information:**

The online version contains supplementary material available at 10.1186/s13052-024-01775-x.

## Introduction

Nephrotic syndrome in pediatric patients presents a complex interplay of symptoms and systemic effects, among which proteinuria and abnormal blood pressure patterns are particularly significant. This condition can profoundly affect pediatric patients’ overall health, impacting their kidney function and cardiovascular health [1]. Children with nephrotic syndrome can benefit greatly from ambulatory blood pressure monitoring (ABPM), which has become an essential tool for evaluating blood pressure patterns. Since ABPM records blood pressure at night and can spot irregular trends, it offers a more thorough picture of the patient’s blood pressure profile than standard blood pressure readings [2].

Nephrotic syndrome results from massive filtration of proteins through glomerular membrane that results in generalized edema and hypovolemia [3, 4], So it was previously thought that most of patients with nephrotic syndrome are known to have normal blood pressure or even hypotensive, but recently many children with nephrotic syndrome have hypertension and American heart association considered nephrotic syndrome as moderate cardiovascular risk factor due to many factors as medications and progression of the renal disease [5, 6].

One of those cardiovascular complications of nephrotic syndrome is hypertension including masked hypertension which is not detected on usual regular follow up and it has harmfull effect on the cardiac condition of the patients [7]. The gold standard for identifying hypertension and evaluating 24-hour blood pressure is ambulatory blood pressure monitoring (ABPM). It also offers information on a number of crucial factors that are not available with other methods of blood pressure measurement, such as masked hypertension [8].

In this study we aimed to assess blood pressure status in children with nephrotic syndrome using ambulatory blood pressure monitoring and its impact on the cardiac condition of those children.

## Patients and methods

We performed a comparative cross-sectional study on pediatric patients with nephrotic syndrome who were following up at Cairo University Children’s Hospital’s nephrology clinic. The selected 90 patients were both male and female, with ages ranging from 5 to 15 years. The patients were diagnosed as nephrotic syndrome from at-least 3months, either steroid-dependent nephrotic syndrome, characterized by two or more relapses in a row during tapering or within 14 days of quitting the medication. Or steroid-resistant nephrotic syndrome, which is characterized by the failure to achieve full remission following four weeks of daily 60 mg/m2 prednisone medication [9]. The patients with glomerular filtration rate (GFR) < 60 or with any structural heart disease were not included in the study.

The following was applied to each patient:

- Detailed medical history including demographic data, onset of the disease, complications and current medications including immunosuppressive and antihypertensive medications.
- Detailed clinical examination was done including: Anthropometric examination (height, weight, body mass index (BMI)), vital signs including blood pressure measurement at the clinic. Blood pressure above the 95th percentile for age, sex, weight, height and BMI was known as high blood pressure. High blood pressure at the clinic with normal ambulatory blood pressure was called white coat hypertension. BMI was calculated according to the equation weight in kg/ height in m^2^.Percentiles with reference to age, sex, weight, height and BMI was calculated.Percentiles < 5 was underweight, percentiles > = 5 and < 85 was healthy weight, percentile > = 85 and < 95 was overweight and percentile > = 95 was obesity [10].
- Investigations including: complete blood picture, electrolytes, kidney functions, serum albumin, cholesterol and urine protein creatinine ratio.
- Ambulatory blood pressure monitoring (ABPM) for 24 h:

The ABPM machine used was Meditech Ambulatory Blood pressure and electrocardiogram (ECG) monitors, made in Hungary. Patient and guardian were counseled not to remove the device and if absolutely necessary, remove it immediately after the reading and put it back on before the next reading.

We looked over the patient’s medical history before starting ABPM to rule out any conditions like latex allergy, clotting issues, or severe nocturnal enuresis (which could get the device wet) [11]. The average arterial pressure during one cardiac cycle, including systole and diastole, is known as mean arterial pressure (MAP). Systemic vascular resistance and cardiac output both have an impact on MAP, and they are both controlled by a number of factors. Elevated blood pressure was defined as the mean ambulatory blood pressure over the age and gender 95th percentiles. Systolic and diastolic pressure differences were used to compute the 24-hour pulse pressure. Published references were used to determine that values more than the 95th percentile were indicative of hypertension [12]. A 10–20% drop in blood pressure from day to night is indicative of normal variance in blood pressure. Those who experience this drop in blood pressure at night are referred to as dippers, and individuals who do not experience a nocturnal drop of between 10 and 20% in mean nighttime systolic blood pressure relative to mean daytime systolic blood pressure are classified as non-dippers [13]. Patients were classified as having ambulatory hypertension if they showed clinic hypertension (clinic blood pressure > 95th percentile), elevated mean ambulatory systolic blood pressure (> 95th percentile) and systolic blood pressure load (> 25%). Masked hypertension (MH) is diagnosed when the clinic BP is normal but ABP is elevated [14].The patients were classified as having normal blood pressure, white coat hypertension, masked hypertension, ambulatory hypertension, or well controlled hypertension based on the percentiles of blood pressure according to age, weight, height, and BMI. Blood pressure below the 95th percentile for age and sex after lifestyle modifications and anti-hypertensive drugs is known as controlled hypertension [6].

Echocardiography: Echocardiography was done at the cardiology clinic of Cairo University Children Hospital. General Electric Vivid 7 served as the ECHO device. Motion mode (M-mode): Conventional echocardiographic parameters, LV posterior and septal wall thickness, diastolic function evaluation, and left ventricular fraction shortening (FS%) and ejection fraction (EF%) were measured and averaged over three cardiac cycles using M-mode echocardiography in accordance with the Teichholz method. Z score was the data’s expression.

Sample size: Using Clinical sample size calculator for analytic study; with 0.05 alpha error and power of the study 0.80. According to literature Ambulatory HTN in a study was present in 33.3% of NS patients. In Pediatric patients with steroid resistant NS nocturnal BP dipping was absent in 67.6%. Prevalence of HTN at presentation in steroid-resistant and congenital NS to range from 10.2% in children < 3 months to 27.9% in adolescents. The minimum sample size calculated is 90 patients [15, 16].

### Statistical design

Using an IBM compatible computer, SPSS (statistical software for social science) version 26.0 was used to tabulate and analyze the acquired data. Two types of statistical analysis were done: Descriptive statistics e.g., g. Number (No), percentage (%), for qualitative data and mean ± SD for quantitative data. and Analytical statistics, such as these: An analysis of the relationship between two or more qualitative variables is done using a parametric test called the chi-squared test (χ2). Student’s t test: a parametric test using normally distributed data that compares two quantitative variables. A non-parametric test called the Mann-Whitney-U is used to compare two quantitative variables when the data are not regularly distributed. When comparing more than two quantitative variables One-Way-ANOVA test is employed. *P*-value of < 0.05 was considered statistically significant.

## Results

Over the course of six months, from December 2022 to June 2023, 90 children with nephrotic syndrome participated in this cross-sectional study.

**Fig. 1:**
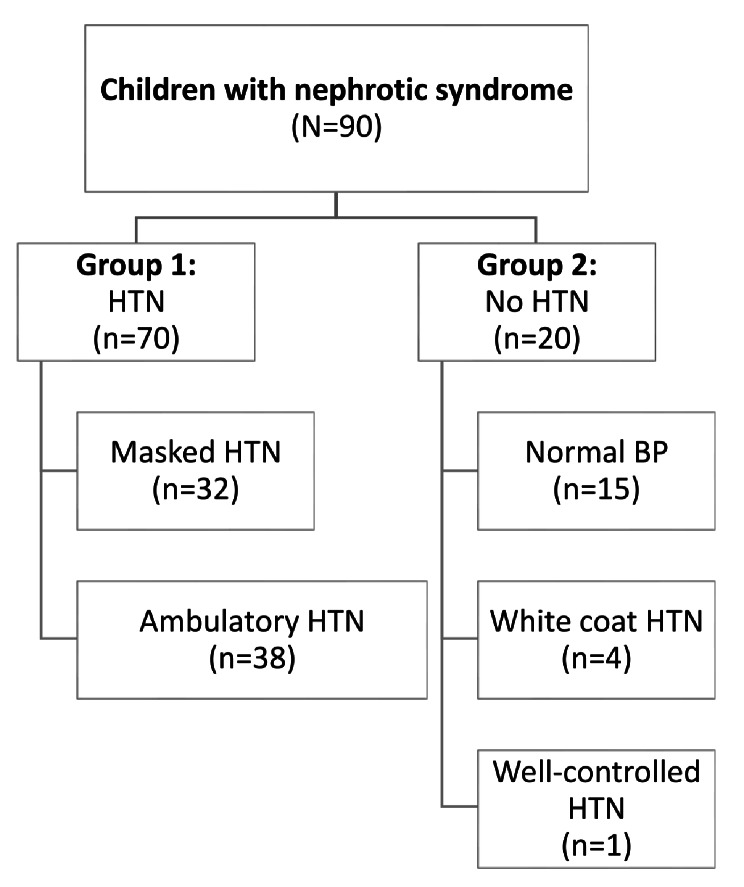
Flow chart of the studied groups

The included patients were divided into two groups: Group1 (*n* = 70): HTN group included masked and ambulatory hypertension, and Group 2 (*n* = 20): non-HTN group included normal blood pressure, white coat HTN and well controlled HTN as shown in Fig. 1.

Interestingly, 35% of the studied cohort (*n* = 32/90) had masked HTN.

The overall z-score of BMI of group 1 is significantly higher than group 2 with *p*-value: 0.029 as we noticed that most hypertensive patients were normal BMI except 2 patients who were obese, while the non-hypertensive group had no obese patients as demonstrated in Table 1.

**Table 1:** Baseline demographic and clinical characteristics of the studied groups (*N* = 90)

		HTN (*n* = 70)	No HTN (*n* = 20)	*P* value
Age (Y)	Mean ± SD	8.8 ± 2.6	8.2 ± 2.2	0.267^*^
Duration of the disease	Mean ± SD	3.0 ± 2.1	2.8 ± 1.9	0.782^@^
Gender	Male	56 (80%)	19 (95%)	0.112^#^
	Female	14 (20%)	1 (5%)	
Weight (Kg)	Mean ± SD	21.3 ± 4.4	19.9 ± 4.1	0.187^*^
Weight SDS for age (Percentile)	Mean ± SD	2.8 ± 3.1	1.8 ± 1.5	0.182^@^
Z score for weight	Mean ± SD	-2.2 ± 0.6	-2.2 ± 0.4	0.667^@^
Height (cm)	Mean ± SD	119 ± 9.0	116.7 ± 9.2	0.325^*^
Height SDS for age (Percentile)	Mean ± SD	5.0 ± 5.3	4.2 ± 3.4	0.569^@^
Z score for height	Mean ± SD	-2.1 ± 0.9	-1.9 ± 0.6	0.471^@^
Interpretation of Z score for height	Normal	29 (41.4%)	9 (45%)	0.775^#^
	Short stature	41 (58.6%)	11 (55%)	
BMI (kg/m^2^)	Mean ± SD	14.9 ± 1.2	14.4 ± 1.0	0.126^*^
BMI SDS for age (Percentile)	Mean ± SD	16.5 ± 11.3	12.0 ± 10.9	0.121^@^
Z score for BMI	Mean ± SD	-1.2 ± 0.7	-1.4 ± 0.7	0.153^@^
Interpretation of Z score for BMI	Underweight	12 (17.1%)	9 (45%)	**0.029** ^**#**^
	Healthy weight	56 (80%)	11 (55%)	
	Overweight	2 (2.9%)	0	
NS type	SDNS	34 (48.6%)	11 (55%)	0.612^#^
	SRNS	36 (51.4%)	9 (45%)	

#: chi^2^ test @: Mann-Whitney U test * Student’s t test

BMI Body mass index, HTN Hypertension, NS Nephrotic Syndrome, SDS Standard deviation score, SDNS Steroid dependent Nephrotic syndrome, SRNS Steroid resistant Nephrotic syndrome

### Blood pressure characteristics among the study group

Table 2 showed that all the overall, morning, night, blood pressure measurements were significantly higher in the hypertensive group, *p* < 0.001. The table also shows that all the MAP, PP measurements, and blood pressure high readings were significantly higher in the hypertensive group, *p* < 0.001. while there was no significant difference between the two groups regarding the pulse average overall, SBP D/N index or DBP D/N index, *p* > 0.05.

**Table 2:** Overall blood pressure characteristics of the studied group (*N* = 90)

		HTN (*n* = 70)	No HTN (*n* = 20)	*P* value
SBP maximum limit value overall (mmHg)	Mean ± SD	127 ± 11.4	103.3 ± 6.3	< 0.001*
DBP maximum limit value overall (mmHg)	Mean ± SD	81.3 ± 10.6	65.9 ± 5.6	< 0.001*
SBP percentile for age	Mean ± SD	99 ± 1.9	77.5 ± 14.9	< 0.001*
DBP percentile for age	Mean ± SD	95.6 ± 5.5	78.5 ± 12.8	< 0.001*
MAP average value overall	Mean ± SD	96.6 ± 10.3	78.4 ± 5.8	< 0.001*
Interpretation	Normal	23 (32.9%)	20 (100%)	**< 0.001** ^**#**^
	Hypertension	47 (67.1%)	0	
PP average value overall	Mean ± SD	45.2 ± 6.4	37.3 ± 2.6	< 0.001*
Pulse average value overall	Mean ± SD	87.4 ± 15.2	92.2 ± 12.2	0.202^*^
SBP maximum limit value during morning (mmHg)	Mean ± SD	130.8 ± 13.3	105 ± 6.1	< 0.001*
DBP maximum limit value during morning (mmHg)	Mean ± SD	85.7 ± 10.5	68.1 ± 5.9	< 0.001*
SBP during morning percentile for age	Mean ± SD	99 ± 4.3	80.5 ± 13.8	< 0.001*
DBP during morning percentile for age	Mean ± SD	97.9 ± 3.3	82.9 ± 12.7	< 0.001*
SBP maximum limit value during night (mmHg)	Mean ± SD	121.9 ± 14.1	102 ± 8.6	< 0.001*
DBP maximum limit value during night (mmHg)	Mean ± SD	76.8 ± 12	63.2 ± 7.1	< 0.001*
SBP during night percentile for age	Mean ± SD	95.8 ± 7.2	71.2 ± 17.8	< 0.001*
DBP during night percentile for age	Mean ± SD	89.6 ± 12.7	69.6 ± 16.9	< 0.001*
MAP average value at daytime	Mean ± SD	100.2 ± 11	80 ± 5.5	< 0.001*
Interpretation	Normal	9 (12.9%)	20 (100%)	**< 0.001** ^**#**^
	Hypertension	61 (87.1%)	0	
MAP average value at night	Mean ± SD	91.9 ± 12.4	76.4 ± 7.5	< 0.001*
Interpretation	Normal	37 (52.9%)	20 (100%)	**< 0.001** ^**#**^
	Hypertension	33 (47.1%)	0	
SBP high reading	Mean ± SD	15 ± 12.1	1.3 ± 1.2	**< 0.001** ^**@**^
DBP high reading	Mean ± SD	20.6 ± 14.3	4.8 ± 3.6	**< 0.001** ^**@**^
SBP D/N index	Mean ± SD	6.1 ± 8.1	2.7 ± 5.9	0.089^@^
DBP D/N index	Mean ± SD	9.0 ± 10.2	6.5 ± 8.8	0.305^@^
SBP night dipping	Yes	18 (25.7%)	1 (5%)	**0.045**#
	No	52 (74.3%)	19 (95%)	
DBP night dipping	Yes	32 (45.7%)	8 (40%)	0.650#
	No	38 (54.3%)	12 (60%)	

#: chi2 test * student’s t test

DBP Diastolic blood pressure, HTN hypertension, MAP Mean arterial pressure, PP Pulse pressure, SBP Systolic blood pressure, SD Standard deviation, D/N day/ night

Most of hypertensive patients were SBP non-dippers, with a significant difference with the non-hypertensive group, *p* = 0.045, while there was no significant difference between the two groups regarding the rate of DBP night dipping, *p* > 0.05. We noticed that the patient who has systolic dipping in non-HTN group is the patient with controlled HTN.

### Antihypertensive medications

Angiotensin converting enzyme inhibitors were taken by 18 (90%) of the non-HTN group as antiprotienuric and by fifty-eight (82.9%) of the HTN group. Among the HTN group, eight children (11.4%) took calcium channel blockers, whereas none of the non-HTN group did. spironolactone was taken by 10 (14.3%) of the HTN group and by 2 (10%) of the non-HTN group.

### Risk factors of hypertension

#### Clinical

The overall z-score of BMI of group 1 is significantly higher than group 2 with *p*-value: 0.029 as shown in Table 1.

None of the patients of group 2 (the non-hypertensive group) were found to have pleural effusion or ascites, but the difference between the two groups were non-significant *p*–values were 0.091 and 0.054 respectively.

#### Laboratory

It was noticed that the serum urea is significantly higher in HTN group than non-HTN group with *p*-value: 0.047, while the serum albumin is significantly lower in HTN group than non-HTN group with *p*-value: 0.017 as demonstrated in Table 3. It was found that at a cut-off point of 1.85, the sensitivity and specificity of serum albumin to predict the occurrence of hypertension in NS patients was 70% and 20% respectively.

**Table 3:** Laboratory characteristics of the studied group (*N* = 90)

		HTN (*n* = 70)	No HTN (*n* = 20)	*P* value
TLC (**×**10^3^)/mm	Mean ± SD	9.9 ± 3.6	8.2 ± 3.3	0.055*
Hb(gm/dl)	Mean ± SD	12.4 ± 1.7	12.0 ± 1.5	0.358^*^
Platelets (**×**10^3^)/mm	Mean ± SD	413.9 ± 149.4	360.3 ± 99.9	0.135^@^
Urea (mg/dl)	Mean ± SD	23.8 ± 22.0	13.7 ± 6.1	**0.047** ^@^
Creatinine (mg/dl)	Mean ± SD	0.6 ± 0.5	0.5 ± 0.2	0.301^@^
Na(mmol/L)	Mean ± SD	139.1 ± 4.2	140.3 ± 3.9	0.248^*^
K(mmol/L)	Mean ± SD	4.3 ± 0.6	4.4 ± 0.5	0.311^*^
Albumin(gm/dl)	Mean ± SD	2.8 ± 1.2	3.5 ± 1.3	**0.017** ^@^
Cholesterol (mg/dl)	Mean ± SD	319.9 ± 135.1	267.4 ± 125.1	0.123^@^
ACR(mg/gm)	Mean ± SD	6378.2 ± 7335	4296.3 ± 4520	0.232^@^
ACR interpretation	No albuminuria	5 (7.1%)	5 (25%)	0.074^#^
	Microalbuminuria	5 (7.1%)	1 (5%)	
	Macroalbuminuria sub nephrotic range.	15 (21.4%)	1 (5%)	
	Nephrotic range macroalbuminuria	45 (64.3%)	13 (65%)	
Pus in urine	Normal	35 (50%)	17 (85%)	**0.005** ^#^
	High	35 (50%)	3 (15%)	
RBCs in urine	Normal	49 (70%)	10 (50%)	0.097^#^
	High	21 (30%)	10 (50%)	
Cast in urine	Nil	39 (55.7%)	20 (100%)	**0.004** ^#^
	Granular cast	25 (35.7%)	0	
	Hyaline cast	5 (7.1%)	0	
	Both granular and hyaline cast	1 (1.4%)	0	
Albumin in urine	Nil	7 (10%)	4 (20%)	0.329^#^
	Trace	3 (4.3%)	2 (10%)	
	1+	6 (8.6%)	4 (20%)	
	2+	27 (38.6%)	5 (25%)	
	3+	26 (37.1%)	5 (25%)	
	4+	1 (1.4%)	0	
NS status	Remission	10 (14.3%)	6 (30%)	0.105
	Activity	60 (85.7%)	14 (70%)	

#: chi^2^ test @: Mann-Whitney U test * Student’s t test

ACR albumin: creatinine ratio in urine, Hb hemoglobin, HTN hypertension, K potassium, Na Sodium, RBC red blood cells, TLC: total leucocyte count

**Fig. 2:**
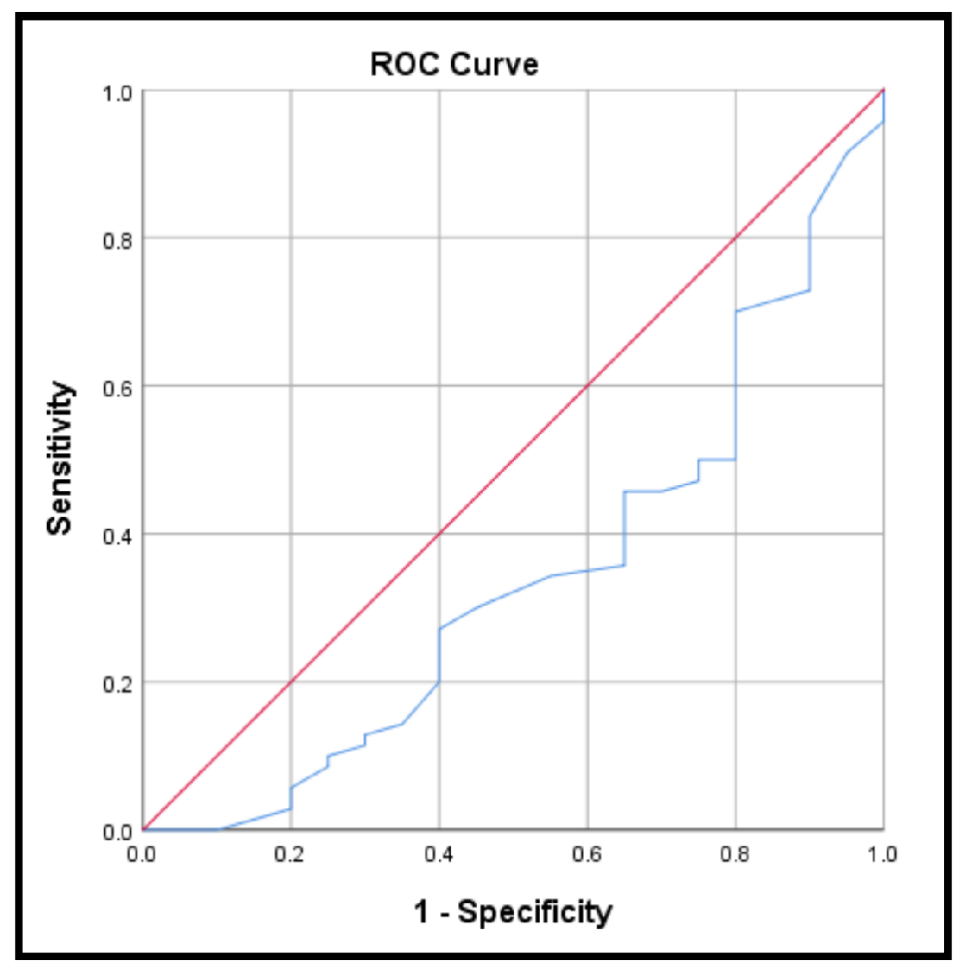
ROC curve for the accuracy of serum urea to predict the occurrence of hypertension in NS patients

It was observed that at a cut-off point of 9.9, the sensitivity and specificity of serum urea to predict the occurrence of hypertension in NS patients was 92.9% and 35% respectively, with *p*-value : 0.024 and 95% CI (0.534–0.798) as shown in Fig. 2.

Regarding the urine it was observed that the degree of albuminuria is more severe in HTN group than non-HTN group but the difference is non-significant (*p*-value: 0.232) and presence of urinary sediments and casts is significantly present in HTN group when compared to non-HTN group (*p*-value: 0.004).which indirectly indicates more severe disease associated with hypertension including masked hypertension.

Group 1 had higher serum cholesterol than Group 2, however the difference was not statistically significant (*p*-value = 0.123).

There was a significant negative correlations between the serum albumin and SBP percentile for age (*p*: 0.017, r: -0.250) and DBP percentile for age (*p*: 0.008, r: -0.277).

Among group 1, it was noticed that 74% (*n* = 52/70) of them were systolic non-dipper, also it was observed that the mean serum K and cholesterol were significantly higher among systolic non-dipper when compared with systolic dipper patients with *p*-values: 0.045 and 0.005 respectively.

### Medications

While the HTN group’s mean steroid dose (1.55 ± 0.48) was lower than the non-HTN group’s (1.62 ± 0.51), the difference was not statistically significant (*p*-value: 0.576).

Tacrolimus was administered as an immunosuppressive medication to the HTN group whereas it was not given to the non-HTN group; nonetheless, the difference between the two groups’ experiences is not statistically significant (*p*-value: 0.219).

### Complications of hypertension

It was observed that the z score of IVS is significantly higher in group 1 (2.5 ± 1.2) when compared to group 2 (1.7 ± 2.1) with *p*-value: 0.025, and that group 1 had a near-significantly greater frequency of LVH (*p*-value of 0.054). It was observed that at a cut-off point of 1.47, the sensitivity and specificity of IVS z score to predict the occurrence of hypertension in NS patients was 84% and 45% respectively.

There were significant positive correlations between the z score of IVS and SBP percentile for age (*p*p: 0.045, r: 0.21) and DBP percentile for age (*p*: 0.043, r: 0.21).

**Fig. 3:**
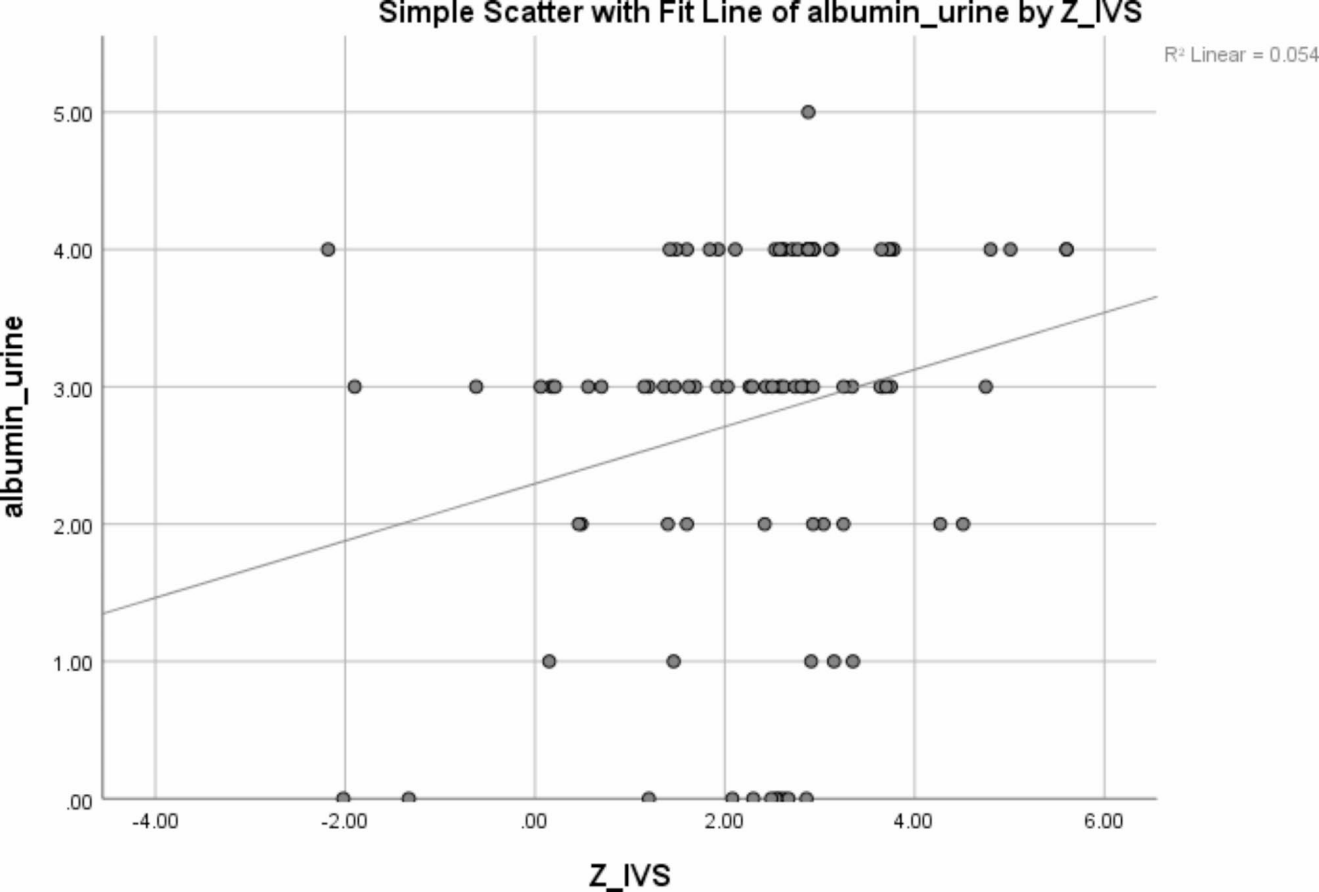
Scatter plot displaying the correlation between the albumin in urine and IVS z score of the studied group

Significant positive correlations were observed between the IVS z score and the albumin creatinine ratio (*p*: 0.028, r: 0.232) as shown in Fig. 3, but non-significant correlations were found between the IVS score and serum albumin (*p*: 0.599, r: 0.056) and BMI (*p*: 0.68, r: -0.055).

It was observed that at a cut-off point of 1.47, the sensitivity and specificity of IVS z score to predict the occurrence of hypertension in NS patients was 84% and 45% respectively with *p*-value : 0.060 and 95%CI (0.481–0.794).

It was noticed that the Z score of IVS is significantly higher among systolic non-dipper (2.7 ± 1.0) when compared to systolic dipper (1.9 ± 1.6), among hypertensive group (group 1) with *p*-value 0.017.

## Discussion

The present work showed that about two thirds (*n* = 70/90) of the children with nephrotic syndrome had either ambulatory (*n* = 38/90) or masked hypertension (*n* = 32/90).

In addition to higher serum urea and lower serum albumin levels being significantly associated with hypertension in children with nephrotic syndrome, increased BMI was found to be associated with hypertension in such children.

Children with nephrotic syndrome who were also hypertensive also had evidence of LVH and a significantly higher IVS z score. This highlights how crucial it is to use ABPM in children with nephrotic syndrome in order to improve their prognosis and reduce long-term morbidities, particularly those related to the cardiovascular system.

Manasa and others found that the prevalence of hypertension is more in steroid dependent nephrotic syndrome than steroid resistant NS which is not detected in our study as there is no significant difference between SRNS and SDNS either males or females in ours [17].

Analyzing the blood pressure characteristics in the studied group yields several noteworthy insights. Firstly, between the two groups, all the overall, morning, night, blood pressure measurements were significantly higher in the hypertensive group, *p* < 0.001, all the MAP, PP measurements, and blood pressure high readings were significantly higher in the hypertensive group, *p* < 0.001. while there was no significant difference between the two groups regarding the pulse average overall, SBP D/N index or DBP D/N index, *p* > 0.05. This significant difference is a clear indicator of the heightened cardiovascular risk in the hypertensive cohort. Moreover, the SBP and DBP percentiles for age also showed significant differences, with the HTN group having higher mean percentiles (SBP: 99 ± 1.9, DBP: 95.6 ± 5.5) than the non-HTN group (SBP: 77.5 ± 14.9, DBP: 78.5 ± 12.8), *p*-values < 0.001. These findings suggest a pronounced deviation from the normal blood pressure range in the hypertensive group, emphasizing the severity of hypertension in these patients and the persistence of hypertension throughout the day, with no significant reduction during nocturnal periods, which is often expected in healthy individuals. All of that highlighting the need for meticulous blood pressure monitoring and management in patients with nephrotic syndrome [18].

Most of hypertensive patients were SBP non-dippers (74.3% of hypertensive group), with a significant difference with the non-hypertensive group (*p* = 0.045), indicating a pronounced absence of typical nocturnal SBP reduction which is clinically relevant as it is typically associated with higher cardiovascular risks and may necessitate more aggressive management strategies. While there was no significant difference between the two groups regarding the rate of DBP night dipping, *p* > 0.05. Ninety-nine children with frequently relapsing NS were included in a study that evaluated ABPM; of them, 72 had nocturnal non-dipping, and 55 had a high nocturnal systolic blood pressure load [19]. In a second, smaller group of 21 patients with primary NS, diurnal blood pressure anomalies and a high frequency of overnight hypertension were also seen. Of these individuals, 8 (38%) had daytime hypertension, 13 (62%) had nighttime hypertension, and 13 (62%) did not dip [20]. Similar findings were seen in another study, where 25 out of 37 patients (68%) did not engage in nocturnal dipping; 14 of these patients did not dip for either SBP or BP, and the remaining patients did not dip for SBP alone [21].

Regarding the risk factors of hypertension, clinically, none of the patients of group 2 (the non-hypertensive group) were found to have pleural effusion or ascites, but the difference between the two groups were non-significant *p*–values were 0.091 and 0.054 respectively. The absence of a significant difference could also imply that these complications are more closely related to other aspects of nephrotic syndrome, such as the severity of proteinuria, the degree of hypoalbuminemia which is more prevalence in hypertensive group1, or overall fluid status, rather than the presence of hypertension alone.

The second clinical risk of hypertension in our study that overall z-score of BMI of hypertensive group 1 is significantly higher than non-hypertensive group 2 with *p*-value: 0.029. It contrasts with the Keshri et al. study, which found no discernible variation between the nephrotic children’s BMI and the incidence of hypertension [22].

In children with NS, the impact of steroid therapy on blood pressure varies. Steroid therapy may cause hypertension (HTTN) or worsen pre-existing hypertension (HTTN) in certain people with NS, while high dosage steroid therapy improves blood pressure in other patients after remission [19].

This could be explained by that the patients on steroid therapy that have hypertension developed significant sodium retention, decreased renin and aldosterone levels, and hypervolemia. However, with steroid therapy, patients who are normo- or hypovolemic experience significant diuresis and natriuresis. Complex interactions between hereditary and environmental factors may be the cause of the variability in how steroids affect blood pressure [19].

In addition, in the current study it was observed that, distinctions in mean serum urea and albumin levels, along with the presence of pus and casts in the urine, between the hypertensive (HTN) and non-hypertensive (non-HTN) groups that yield significant insights into the interplay of hypertension and pediatric nephrotic syndrome. The HTN group displayed a higher mean serum urea level (23.8 ± 22) in comparison to the non-HTN group (13.7 ± 6.1), with a statistically significant difference (*p* = 0.047). This may be explained by elevated blood urea despite normal serum creatinine, which is linked to the children’s hypovolemic state. This, in turn, activates the Renin-Angiotensin System (RAS), which causes hypertension in such children [23].

Furthermore, the HTN group exhibited a lower mean serum albumin level (2.8 ± 1.2) than the non-HTN group (3.5 ± 1.3), with a significant *p*-value of 0.017. This implies a more severe protein loss in hypertensive patients, considering hypoalbuminemia as a hallmark of nephrotic syndrome which may be attributed to increased glomerular damage or a more severe manifestation of the disease [24].

Hypoalbuminemia, a hallmark of Nephrotic syndrome, leads to decreased plasma oncotic pressure and subsequent fluid shifts into the interstitial space, causing edema. The body’s compensatory mechanisms, including activation of the renin-angiotensin-aldosterone system, may contribute to elevated blood pressure, particularly in pediatric patients [25]. In our patients, there was a significant negative correlations between the serum albumin and SBP percentile for age (p: 0.017, r: -0.250) and DBP percentile for age (p: 0.008, r: -0.277).

In a study done on 207 hypertensive children, there were 51.21% of cases were non-dippers, total cholesterol level was significantly higher in non-dippers than dippers (4.34 vs. 3.99 mmol/L, *p*-value :0 0.034) [26]. Among our group 1, it was noticed that 74% (*n* = 52/70) of them were systolic non-dipper, also it was observed that the mean serum cholesterol were significantly higher among systolic non-dipper when compared with systolic dipper patients with *p*-value 0.005.

In this work it was observed that the mean serum K was significantly higher among systolic non-dipper when compared with systolic dipper patients with *p*-value: 0.045. which do not agree with a study in adult patients that showed serum K was significantly higher among systolic dipper when compared to systolic non-dipper [27].

It was observed that the z score of IVS is significantly higher in hypertensive group. The elevated Z score in the hypertensive group could be indicative of early cardiac remodeling, a known complication of sustained hypertension [28].

There were significant positive correlations between the z score of IVS and SBP percentile for age (p: 0.045, r: 0.21) and DBP percentile for age (p: 0.043, r: 0.21). In current study, there is significant positive correlations were observed between the IVS z score and the albumin creatinine ratio (p: 0.028, r: 0.232). Proteinuria may be an important factor in the development of chronic inflammation in NS with subsequent myocardial involvement [29].

In our study, It was noticed that the Z score of IVS is significantly higher among systolic non-dipper (2.7 ± 1.0) when compared to systolic dipper (1.9 ± 1.6), among hypertensive group (group 1) with *p*-value 0.017. Patients without dipper patterns are more likely to experience end-organ damage in essential pediatric hypertension, LV hypertrophy, and early LV dysfunction. This is important since it can lead to unfavorable cardiac remodeling [29].

The study’s limitation was the requirement for a larger cohort of children and for follow-up on the children’s blood pressure, cardiac health, and nephrotic syndrome status, including whether there was activity or remission during the follow-up period. Additionally, the dosage of steroids given to the children will be correlated to these variables. We recommend further studies on Control of masked hypertension in children with nephrotic syndrome and its effects on IVS hypertrophy.

Finally, the present study examined the diagnostic accuracy of BMI, IVS z score, serum urea, and serum albumin to predict the occurrence of hypertension in pediatric nephrotic syndrome patients. The results suggested that serum urea has the highest sensitivity (92.9%) for predicting hypertension (AUC = 0.666), while BMI and IVS z score demonstrate moderate diagnostic accuracy (AUC = 0.626 and 0.638, respectively). In contrast, serum albumin shows limited utility in this prediction, as indicated by its lower AUC value (0.340). Overall, this study adds valuable evidence to the existing knowledge on pediatric nephrotic syndrome, particularly concerning the interrelationship between proteinuria, ambulatory blood pressure, and various demographic and clinical factors. The findings will help guide future research and clinical practice to characterize risk factors better, understand underlying mechanisms, formulate management strategies, and ultimately improve outcomes for this vulnerable patient population.

## Conclusions

Children with nephrotic syndrome are particularly vulnerable to experience ambulatory hypertension and masked hypertension, which may adversely impact their cardiac condition in the form of affection of IVS, because they are not detectable by standard blood pressure readings at the clinic.

## Electronic supplementary material

Below is the link to the electronic supplementary material.

Supplementary Material 1

## Data Availability

The datasets used and/or analysed during the current study are available from the corresponding author on reasonable request.

## Abbreviations

ABPM: Ambulatory blood pressure monitoring
ACR: Albumin creatinine ratio in urine
BMI: Body mass index
BP: Blood pressure
CUCH: Cairo University Children Hospital
D/N: Day/Night
DBP: Diastolic blood pressure
ECG: Electrocardiogram
GFR: Glomerular filtration rate
Hb: Hemoglobin
HTN: Hypertension
IVS: Interventricular septum
K: Potassium
MAP: Mean arterial pressure
MH: Masked hypertension
Na: Sodium
NS: Nephrotic syndrome
PP: Pulse pressure
RBC: Red blood cells
SBP: Systolic blood pressure
SD: Standard deviation
SDNS: Steroid dependent Nephrotic syndrome
SDS: Standard deviation score
SRNS: Steroid resistant Nephrotic syndrome
TLC: Total leucocyte count
