# Supplementary material for: Ambulatory blood pressure monitoring in Egyptian children with nephrotic syndrome: single center experience
Source: Ital J Pediatr. 2024 Oct 12;50:214. doi: 10.1186/s13052-024-01775-x (PMC11470724; doi:10.1186/s13052-024-01775-x)
Supplement: Supplementary file 1 — Supplementary Material 1 [file 13052_2024_1775_MOESM1_ESM.docx]

Dear Editor in Chief,

Thank you for giving us the opportunity to submit a revised draft of the manuscript “Ambulatory blood pressure monitoring in Egyptian children with nephrotic syndrome: single center experience” for publication in the *Italian journal of pediatrics*. We appreciate the time and effort that you and the reviewers dedicated to providing feedback on our manuscript and are grateful for the insightful comments on and valuable improvements to our paper. We have incorporated most of the suggestions made by the reviewers.

***Response to Reviewer's Comments***

| ***Reviewer Comment*** | ***Response*** | ***Changes within the revised manuscript*** |
| --- | --- | --- |
| ***Reviewer 1***  ***Changes within revised version of the manuscript were highlighted in yellow*** | | |
| 1. Although this is a single-center experience report, the authors should consider increasing the sample size to enhance the generalizability of the results. Moreover, considering the heterogeneity of nephrotic syndrome, are the patient subtypes (such as steroid-dependent and steroid-resistant) sufficiently diverse in the study? | Thank you sir for appointing this.  We agree with reviewer regarding the sample size as we mentioned that the need of larger cohort of included patient in study limitation at the last paragraph of discussion.  But actually this sample size is the minimum sample size calculated by the statistics (n=90 patients).  Regarding the patient subtypes ,about 15 -20 % of idiopathic nephrotic syndrome are steroid resistant and from 60-70% are steroid sensitive , 30 % of them will be further classified as having frequently relapsing nephrotic syndrome or steroid-dependent nephrotic syndrome***(Trautmann et.al,2023)***.  So we focused on those two categories of nephrotic syndrome, as they will need long term treatment and follow up.  And clinical hypertension easily detected in the clinic in those children during their follow up, but the problem of missing the masked hypertension (and subsequent complications especially cardiac one) or over estimation in white coat hypertension. Which can detected only by ambulatory blood pressure monitoring.  Trautmann, A., Seide, S., Lipska-Ziętkiewicz, B.S. *et al.* Outcomes of steroid-resistant nephrotic syndrome in children not treated with intensified immunosuppression. *Pediatr Nephrol* **38**, 1499–1511 (2023). <https://doi.org/10.1007/s00467-022-05762-4>. | The study's limitation was the requirement for a larger cohort of children |
| 2. The study mentions the need to consider the impact of the dose of steroids administered to the children on blood pressure and other indicators, but this is not detailed. This could be a significant factor contributing to elevated blood pressure and should be incorporated into the analysis | We appreciate the reviewer’s feedback.  When we compared both groups HTN and non-HTN, we found that the steroid dose is higher in HTN group, but the difference was non –significant as mentioned at the results section.  The variable effect of steroids on BP and explanation are added in discussion section. | Results section :  Medications:  While the HTN group's mean steroid dose (1.55 ± 0.48) was lower than the non-HTN group's (1.62 ± 0.51), the difference was not statistically significant (p-value: 0.576).  Discussion section:  In children with NS, the impact of steroid therapy on blood pressure varies. Steroid therapy may cause hypertension (HTTN) or worsen pre-existing hypertension (HTTN) in certain people with NS, while high dosage steroid therapy improves blood pressure in other patients after remission.  This could be explained by that the patients on steroid therapy that have hypertension developed significant sodium retention, decreased renin and aldosterone levels, and hypervolemia. However, with steroid therapy, patients who are normo- or hypovolemic experience significant diuresis and natriuresis. Complex interactions between hereditary and environmental factors may be the cause of the variability in how steroids affect blood pressure. |
| 3. The conclusions mention the diagnostic accuracy of BMI, IVS z-score, serum urea, and serum albumin for predicting hypertension. Although serum urea shows high sensitivity, the AUC value is not high, suggesting limited predictive ability. The authors should discuss the clinical significance and limitations of these findings. | Despite all diagnostic factors showing statistically significant p-values, the ROC curve analysis presents a more detailed picture of their diagnostic performance. Specifically: Serum urea demonstrated high sensitivity (92.9%) but had a low AUC. This indicates that while serum urea is effective at detecting the hypertension in children with nephrotic syndrome when it is present, its overall ability to discriminate between hypertensive and non-hypertensive cases is limited. This limited discrimination capability is reflected in its low specificity, which can lead to a higher rate of false positives.Other diagnostic tests showed varying levels of sensitivity and specificity:Sensitivity: 84% and 70%Specificity: 45% and 20% for IVS z score and serum albumin respectively.These values reveal that while these tests have moderate to high sensitivity, indicating their ability to detect hypetension when it is present, their low specificity suggests a high rate of false positives. This implies that these findings are not very effective at accurately identifying hypertensive individuals.Overall, the combination of high sensitivity for serum urea and moderate sensitivities for serum albumin and IVSz score, along with their low specificities, suggests that these diagnostic tests, while significant, are not highly effective at distinguishing between hypertensive and non-hypertensive. This underscores the need for a multi-faceted diagnostic approach that combines various tests to improve overall accuracy and reduce misclassification. This analysis highlights that statistical significance alone does not ensure high diagnostic performance, and integrating multiple diagnostic indicators is crucial for accurate disease identification.  The accuracy of serum albumin and IVS Z score are added at results section. | Results section :  It was found that at a cut-off point of 1.85, the sensitivity and specificity of serum albumin to predict the occurrence of hypertension in NS patients was 70 % and 20 % respectively.  It was observed that at a cut-off point of 1.47, the sensitivity and specificity of IVS z score to predict the occurrence of hypertension in NS patients was 84 % and 45 % respectively. |
| 4. The conclusion of the article's abstract section seems to be inconsistent with the conclusion in the discussion section; please note that and the authors need to revise it. | We agree with the reviewer and we are apologize for this typing error.  The conclusion part in manuscript is added in the last paragraph of discussion section and removed from conclusion section . | Discussion section :  Finally, the present study examined the diagnostic accuracy of BMI, IVS z score, serum urea, and serum albumin to predict the occurrence of hypertension in pediatric nephrotic syndrome patients. The results suggested that serum urea has the highest sensitivity (92.9%) for predicting hypertension (AUC= 0.666), while BMI and IVS z score demonstrate moderate diagnostic accuracy (AUC= 0.626 and 0.638, respectively). In contrast, serum albumin shows limited utility in this prediction, as indicated by its lower AUC value (0.340). Overall, this study adds valuable evidence to the existing knowledge on pediatric nephrotic syndrome, particularly concerning the interrelationship between proteinuria, ambulatory blood pressure, and various demographic and clinical factors. The findings will help guide future research and clinical practice to characterize risk factors better, understand underlying mechanisms, formulate management strategies, and ultimately improve outcomes for this vulnerable patient population.  Conclusion section :  Children with nephrotic syndrome are particularly vulnerable to experience ambulatory hypertension and masked hypertension, which may adversely impact their cardiac condition in the form of affection of IVS, because they are not detectable by standard blood pressure readings at the clinic. |
| ***Reviewer 2***  ***Changes within revised version of the manuscript were highlighted in green*** | | |
| sample size calculation should be added | We appreciate the reviewer’s feedback.  The sample size is added in patients and methods section | Patients and methods:  Using Clinical sample size calculator for analytic study; with 0.05 alpha error and power of the study 0.80. According to literature Ambulatory HTN in a study was present in 33.3% of NS patients. In Pediatric patients with steroid resistant NS nocturnal BP dipping was absent in 67.6%. Prevalence of HTN at presentation in steroid-resistant and congenital NS to range from 10.2% in children <3 months to 27.9% in adolescents. The minimum sample size calculated is 90 patients |
| - future direction -  Control of HT in masked HT NS pts and its effects on IVS hypertrophy would be the future direction | Thank you sir for appointing this.  We agree with the reviewer and added in recommendation at the discussion section. | Discussion section:  We recommend further studies on Control of masked hypertension in children with nephrotic syndrome and its effects on IVS hypertrophy. |
